# Plastic Polymers and Antibiotic Resistance in an Antarctic Environment (Ross Sea): Are We Revealing the Tip of an Iceberg?

**DOI:** 10.3390/microorganisms12102083

**Published:** 2024-10-17

**Authors:** Gabriella Caruso, Maurizio Azzaro, Ombretta Dell’Acqua, Maria Papale, Angelina Lo Giudice, Pasqualina Laganà

**Affiliations:** 1 Institute of Polar Sciences, National Research Council, Spianata S. Raineri 86, 98122 Messina, Italy; maurizio.azzaro@cnr.it (M.A.); maria.papale@cnr.it (M.P.); angelina.logiudice@cnr.it (A.L.G.); 2 Department of Earth, Environment and Life Sciences (DISTAV), University of Genoa, Corso Europa 26, 16132 Genoa, Italy; ombretta.dellacqua@gmail.com; 3 Italian Collection of Antarctic Bacteria, National Antarctic Museum (CIBAN-MNA), Viale Ferdinando Stagno d’Alcontrès 31, 98168 Messina, Italy; 4 Department of Biomedical and Dentistry Sciences and Morphological and Functional Images (BIOMORF), University of Messina, Via Consolare Valeria 1, 98125 Messina, Italy; plagana@unime.it

**Keywords:** microbial biofilm, plastic pollution, antibiotic-resistant bacteria, Ross Sea, Antarctica

## Abstract

Microbial colonization of plastic polymers in Antarctic environments is an under-investigated issue. While several studies are documenting the spread of plastic pollution in the Ross Sea, whether the formation of a plastisphere (namely the complex microbial assemblage colonizing plastics) may favor the spread of antibiotic-resistant bacteria (ARB) in this marine environment is unknown yet. A colonization experiment was performed in this ecosystem, aiming at exploring the potential role of plastic polymers as a reservoir of antibiotic resistance. To this end, the biofilm-producing activity and the antibiotic susceptibility profiles of bacterial strains isolated from biofilms colonizing submerged polyvinylchloride and polyethylene panels were screened. The colonization experiment was carried out at two different sites of the Ross Sea, namely Road Bay and Tethys Bay. Most of bacterial isolates were able to produce biofilm; several multidrug resistances were detected in the bacterial members of biofilms associated to PVC and PE (also named as the plastisphere), as well as in the bacterial strains isolated from the surrounding water. The lowest percentage of ARB was found in the PE-associated plastisphere from the not-impacted (control) Punta Stocchino station, whereas the highest one was detected in the PVC-associated plastisphere from the Tethys Bay station. However, no selective enrichment of ARB in relation to the study sites or to either type of plastic material was observed, suggesting that resistance to antibiotics was a generalized widespread phenomenon. Resistance against to all the three classes of antibiotics assayed in this study (i.e., cell wall antibiotics, nucleic acids, and protein synthesis inhibitors) was observed. The high percentage of bacterial isolates showing resistance in remote environments like Antarctic ones, suffering increasing anthropic pressure, points out an emerging threat with a potential pathogenic risk that needs further deepening studies.

## 1. Introduction

Microbial colonization of plastic polymers commonly occurs in aquatic habitats, resulting in the formation of a new ecological niche, named the “plastisphere”, and biofouling [1,2]. Plastic items serve as a suitable substrate for the adhesion of microorganisms, including pathogens, and organic contaminants [3], but the actual potential risks deriving from adsorbed antibiotics are far from being known yet. The health of Antarctic environments is threatened by increasing release of plastic items and pharmaceutical residues, leading to antimicrobial resistance phenomena; both these are now considered as two major emerging issues impacting on these remote environments [4,5,6,7,8]. Indeed, several Antarctic environmental domains are facing an increasing anthropogenic pressure from scientific research and tourism activities; despite the awareness of these threats, the impacts and the potential risks related to the spread of plastic polymers are, however, not well known yet. Particularly, plastic items are now regarded as potential vectors of contaminants including antibiotics, that may adsorb on their surface and persist for a long time in the environment [3]. The main factors affecting the interaction between microplastics and antibiotics have been identified, such as biofilm growth, plastic aging degree, and polymer type (for plastics) and ionization constant and octanol–water partition coefficient (for antibiotics), but the combined toxicity of microplastics and antibiotics is still almost unknown [9]. The process of plastic colonization by microbial biofilms has been reported to favor the spread of antimicrobial resistance phenomena [9,10,11]. According to the World Health Organization [12], antimicrobial resistance (AMR) is a global concern threat, that may be exacerbated by climate warming and anthropogenic activities, but in Antarctic environments the issue related to the dissemination of antibiotic-resistant bacteria (ARB) and genes (ARGs) through plastic pollution is still an unsolved question. AMR phenomena are not only related to misuse or abuse of antibiotics (commonly indicated as anthropogenic AMR [13]), but also antibiotic resistance determinants are naturally present in microbial communities regardless of human presence (indicated as natural AMR [13], or the environmental antibiotic “resistome” according to Surette and Wright [14]) even in remote pristine Antarctic soils [15].

Due to the limited knowledge about potential links between plastic pollution and antibiotic resistance, further research is needed to identify the natural reservoirs of AMR and the natural/anthropogenic factors potentially involved in the spread of ARB/ARGs across all the environmental matrices. Particularly, remote Antarctic environments, where anthropogenic pressure on these extreme regions is lower compared to other Earth regions, represent suitable models where the complex mechanisms and pathways of acquisition and transmission of AMR can be studied also in an evolutionary perspective. The conventional paradigm of Antarctic ecosystems as pristine environments has recently been fully overcome following the increasing number of reports that have documented the widespread occurrence of ARB in aquatic and terrestrial habitats. Antibiotic residues and ARB/ARGs are directly related to the anthropic presence and reach the marine environment by sewage discharge, posing serious risks to this remote environment; moreover, antibiotics and their residues may persist for several months into the environment, causing threats to microbial communities [16]. In addition, biofilmproduction represents a strategy adopted by microbial communities to overcome stressing conditions; within microbial biofilm consortia, specific antibiotic resistance mechanisms, including the ability of the biofilm matrix to prevent antibiotics from reaching the cells, were found to occur [17].

Recent studies have highlighted that Antarctic environments act as potential reservoirs for antibiotic-resistant bacteria or genes (see for a review [13]), confirming that even this remote region suffers human-related impacts. In a previous study, ARB were isolated from a microbial biofilm colonizing the surface of a plastic item collected from King George Island [18]; multidrug resistance was detected against cephalosporins (cefazolin and cefuroxime), quinolones (cinoxacin), and beta-lactams (ampicillin, mezlocillin, and carbenicillin). The Ross Sea is considered as a hotspot of biodiversity thanks to its variety of habitats, and it represents one of the most productive regions in the Antarctica [19]; ongoing perturbations such as climate change and increasing inputs of pollutants, including antibiotics, are expected to result in unpredictable effects on the natural trophic structure of this environment. Although the Ross Sea was previously known as the last pristine ocean [20], the ecotoxicological effects of such xenobiotic compounds on the marine food web and their fate in this environment are still mostly unknown.There is a limited scientific literature reporting on the occurrence and spread of AMR in the Ross Sea [16]; more specifically, the role of plastic pollution in the spread of ARB or genetic determinants of antibiotic resistance represents an issue not yet estimated in this area. Particularly, questions like: (i) Are plastic polymers (such as polyvinyl chloride–(PVC) and polyethylene (PE)) hosting ARB? (ii) Is the occurrence of ARB differentially favored by the polymeric nature (PVC or PE)? (iii) How do antibiotic susceptibility profiles change in reply to natural or anthropic variables? need to be clarified. Based on the higher number of heterotrophic bacteria colonizing PVC than PE panels found during a previous colonization experiment performed in the Ross Sea [21], our working hypothesis was that the occurrence of biofilm-producing and ARB could be significantly affected by the substrate type. Moreover, two areas of the Ross Sea were investigated, giving us the opportunity to understand whether exposure to different forcings (i.e., a salinity gradient, as a proxy of climate warming, like in Tethys Bay, or the presence of a treated sewage effluent close the research station Mario Zucchelli, like in Road Bay) could affect the antibiotic susceptibility patterns of the bacterial isolates.

## 2. Materials and Methods

### 2.1. Study Area

**Figure 1:**
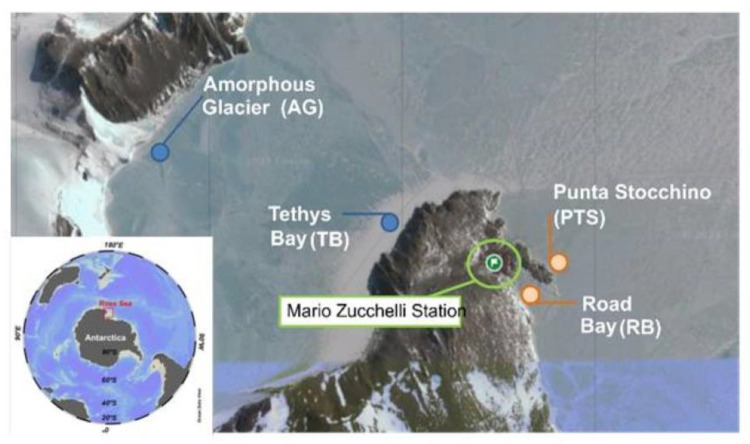
Study sites in the Ross Sea, Antarctica.

For the microbial colonization experiment, PVC and PE panels mounted on stainless structures were immersed at 5 and 20 m depths during the 33rd Italian Antarctic campaign (2017–2018) and left undisturbed in situ for different time periods (3, 9, and 12 months) to assess the biofouling process. The experiment was set up at two sites of Terra Nova Bay, namely Road Bay close to the Zucchelli research station, affected by anthropic activity and by the release of sewage effluent; Tethys Bay, unaffected by human presence, was close to Amorphous Glacier and exposed to a salinity gradient following climate warming (Figure 1). At Tethys Bay, a long-term colonization experiment was performed; there, the experiment lasted 12 months only, while at Road Bay, due to the exposure of this site to human pressure, additional short-term samplings (after immersion for 3 and 9 months) were made. The coordinates of the study sites were as follows: RB (Latitude 74°41.743′ S Longitude 164°07.125′ E), PTS (Latitude 74°41.651′ S Longitude 164°07.303′ E), TB (Latitude 74°41.417′ S Longitude 164°06.303′ E), AG (Latitude 74°41.234′S Longitude 164°02.135′ E).

### 2.2. Biofilm Production Assay

The production of biofilm by bacterial isolates was assayed using the method of crystal violet staining described by O’Toole et al. [22]. Microtiter plates were filled with a bacterial suspension (100 μL, eight wells per strain), while Marine broth (Condalab, Torrejon de Ardox, Madrid, Spain) was used to fill a control well; after incubation at 15 °C for 48 h, the supernatant of each well was removed and three washings with sterile physiological saline (0.9% NaCl) were made. Non-adherent bacteria were removed by strong shaking, followed by staining with 150 μL crystal violet (1% final concentration, Sigma-Aldrich, St. Louis, MO, USA) for 45 min. The excess of stain was removed with treatment with 95% ethanol, and measurements of the optical density (OD) of each well were carried out using a Multiskan GO Microplate Spectrophotometer (Thermo-Fisher Scientific Italia, Rodano, Milan, Italy) at a wavelength of 550 nm.

### 2.3. Isolation and Identification of Bacterial Strains

Microbial biofilms developed on the panels were immediately collected after the recovery of the panels, using a sterile spatula. Serial dilutions of the biofilms in sterile seawater were made, and volumes of 0.1 mL were streaked on Marine agar 2216 plates (Condalab) incubated at +5 °C up to 21 days. Contextually to biofilm samples, seawater samples were collected using a Niskin bottle and 0.1 mL were spread on the same marine agar plates and incubated for the same time interval. The grown colonies were counted and further streaked on the same culture medium until axenic cultures were obtained. The bacterial isolates were first phenotypically characterized [23] and identified by 16S rRNA sequencing. Briefly, amplification of the 16S rRNA gene was performed with a thermocycler (Mastercycler GeneAmp PCR-System 9700, Applied Biosystems, Waltham, MA, USA) using bacteria-specific primers 27F (5′-AGAGTTTGATC(AC)TGGCTCAG–3′) and 1492R (5′-TACGGYTACCTTGTTACGAC-3′). The reaction mixtures were assembled at 0 °C and contained 1 μL DNA, 0.4 μL of each of the two primers (10 μM), 0.4 μL of each dNTP (10 mM), 2 μL of reaction buffer 10×, 0.4 μL of Bovine Serum Albumine (3 mg/mL), 0.2 μL of Taq polymerase 5 PRIME (5 U/μL, Bioline, Heidelberg, Germany), and sterile Milli-Q water to a final volume of 20 μL. Negative controls for DNA extraction and PCR setup (reaction mixture without a DNA template) were also used in every PCR run. The PCR program was as follows: (1) 95 °C for 1.30 min; (2) 5 cycles at 95 °C for 30 s, 60 °C for 30 s, and 72 °C for 4 min; (3) 5 cycles at 95 °C for 30 s, 55 °C for 30 s, and 72 °C for 4 min; (4) 25 cycles at 95 °C for 30 s, 50 °C for 30 s, and 72 °C for 4 min; (5) 72 °C for 10 min; (6) 60 °C for 10 min. The DNA quality was checked by running 2 μL of each sample on 1% agarose gel (*w*/*v*) in TAE buffer 1X (0.04 M Tris-acetate, 0.02 M acetic acid, 0.001 M EDTA) containing SyBr Safe (1 μL on 25 mL final volume, Invitrogen, Waltham, MA, USA). Amplified products and sequencing were carried out at the Eurofins Europe laboratory (Konstanz, Germany). Sequences were read and corrected manually using the software FinchTV version 1.4. Next relatives of isolates were determined by comparison sequences in the NCBI GenBank and the EMBL databases using BLAST, and the “Seqmatch” and “Classifier” programs of the Ribosomal Database Project II (https://github.com/rdpstaff/RDPTools/ accessed on 15 March 2021) [24].

### 2.4. Screening of the Susceptibility to Antibiotics of the Bacterial Isolates

Antibiotic susceptibility profiles were studied according to the plate diffusion Kirby–Bauer [25] method. Briefly, a bacterial suspension at a 0.5 McFarland turbidity standard (BioMérieux, Marcy L’Etoile, France) was prepared in peptone water for each bacterial isolate, corresponding to 1.5 × 10^8^ colony forming units/mL. After streaking of the inoculum on Mueller–Hinton agar (Merck Life Science, Milan, Italy) plates, antibacterial disks (Thermo Fisher Scientific, Rodano, Milan, Italy) were placed on the agar surface to visualize the production of an inhibition halo around each disk. The diameters of the halos were measured using a precision caliper (Mitutoyo, Andover, UK) after incubation for 24–48 h at room temperature. A wide range of antibiotic molecules were assayed, which were distinguished according to their mechanisms of action into three main classes:

(a) Molecules acting on the cell wall: Amoxicillin/Clavulanate (AMC, 30 μg); Amoxicillin (AML, 10 μg); Ampicillin (AMP, 10 μg); Aztreonam (ATM, 30 μg); Carbenicillin (CAR, 100 μg); Cefazolin (KZ, 30 μg); Ceftazidime (CAZ, 30 μg); Cefotaxime (CTX, 30 μg); Ceftriaxone (CRO, 30 μg); Cefuroxime (CXM, 30 μg); Colistin sulfate (CT, 10 μg); Fosfomycin (FOS, 50 μg); Imipenem (IPM, 10 μg); Methicillin (MET, 5 μg); Mezlocillin (MEZ, 10 μg); Oxacillin (OX, 1 μg); Penicillin G (P, 10 μg); Piperacillin (PRL, 100 μg); Cefoxitin (FOX, 30 μg); Teicoplanin (TEC, 30 μg); Teicoplanin (TOB, 30 μg); Vancomycin (VAN, 30 μg);
(b) Molecules that inhibit nucleic acids, including rifamycins, quinolones: Ciprofloxacin (CIP, 5 μg); Levofloxacin (LEV, 5 μg); Nalidixic acid (NA, 30 μg); Nitrofurantoin (F, 300 μg); Norfloxacin (NOR, 10 μg); Ofloxacin (OFX, 5 μg); Pipemidic acid (PIP, 75 μg); Rifampicin (RD, 30 μg);
(c) Molecules that inhibit protein synthesis, including aminoglycosides, tetracyclines; chloramphenicol, macrolides: Amikacin (AK, 30 μg); Azithromycin (AZM, 15 μg); Chloramphenicol (C, 30 μg); Clindamycin (DA, 2 μg); Doxycycline (DO, 30 μg); Erythromycin (E, 15 μg); Gentamicin (CN, 10 μg); Lincomycin (MY, 2 μg); Linezolid (LZD, 10 μg); Minocycline (MH, 30 μg); Mupirocin (MUP, 5 μg); Neomycin (N, 30 IU); Netilmicin (NET, 30 μg); Tetracycline (TE, 30 μg); Tigecycline (TGC, 15 μg).

According to the EUCAST criteria [26], each bacterial isolate was classified as resistant (R), moderately sensitive (I), and sensitive (S). A further category (very sensitive, SS) was also introduced. The antibiotic susceptibility assays were performed under the same experimental conditions and using standard concentrations for the assayed antibiotics; however, as the value of the diameter of inhibition is a qualitative value, in order to make data reciprocally comparable, it was necessary to convert the qualitative value obtained from the measurement of the diameter of the inhibition zone into an arbitrary numerical value according to a range between 0 and 3, as reported in Table 1.

### 2.5. Statistical Analysis of Data

Antibiotic resistance profiles, converted into quantitative data, were averaged per each category of assayed antibiotics and then elaborated by correlating them with the Multiple Antibiotic Resistance (MAR) index values (see below) using the Pearson correlation function available in the Microsoft Excel software program (version 10).

## 3. Results

### 3.1. Ability to Produce Biofilm of Bacterial Isolates

**Figure 2:**
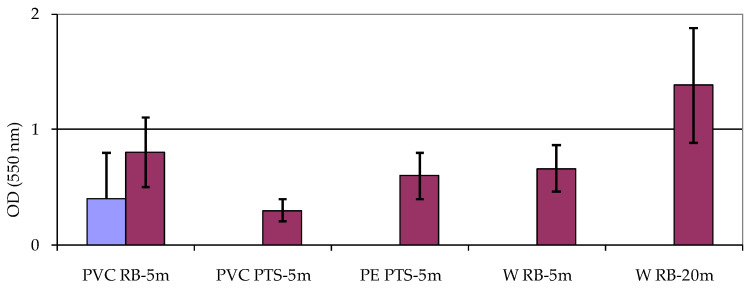
Mean ± standard deviation (from *n* = 3 independent sample measurements) values of Optical Density (OD) recorded in the biofilm production assay. Reported is the biofilm production in selected bacterial strains isolated from different matrices: polyvinylchloride (PVC), polyethylene (PE), and water (W) from Road Bay (RB) and Punta Stocchino (PTS) stations.

The screening of bacterial isolates for biofilm formation yielded the results summarized in Figure 2. All the assayed strains were found positive to produce biofilm, although a great variability in this ability was observed within the strains isolated from PVC-associated biofilm at station RB-5 m and from water collected at station RB-20 m (where OD reached standard deviation values of 0.3 and 0.5, respectively). At the impact station RB, affected by treated wastewater inputs, all the bacteria isolated from PVC showed OD values higher than at the station PTS. Moreover, at RB station, biofilm production on PVC increased from 9 to 12 months. At the control station PTS, microbial colonization was favored more on PE rather than on PVC. Bacterial strains isolated from water showed higher ability to produce biofilm at depths of 20 m compared to those living at 5 m depth.

### 3.2. Screening of Bacterial Isolates for Antibiotic Susceptibility

The average percentages of the observed resistance (R) and sensitivity (S) of the bacterial strains isolated from PVC- and PE-associated biofilms, referred to the total of the isolates, are reported in Table 2. At station RB, in the plastisphere associated to PVC, the sum of the percentages of resistant (R) +intermediate resistant (I) bacteria increased over time, conversely with the trend recorded at station PTS. Surprisingly, the highest percentage of ARB was detected at station TB at the depth of 20 m, while after 12 months of immersion, similarly low percentages of ARB were found at PTS-5 m and AG-20 m stations (characterized by low waste impact and diluted waters due to ice-melting, respectively).

**Figure 3:**
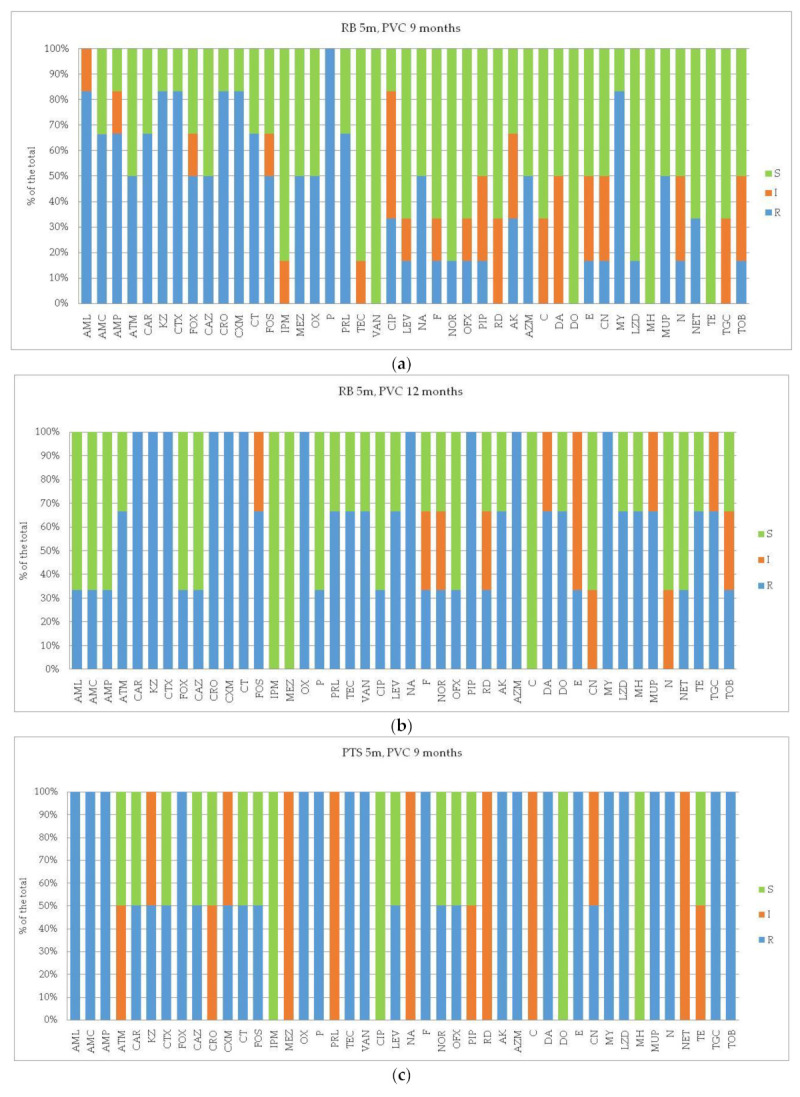

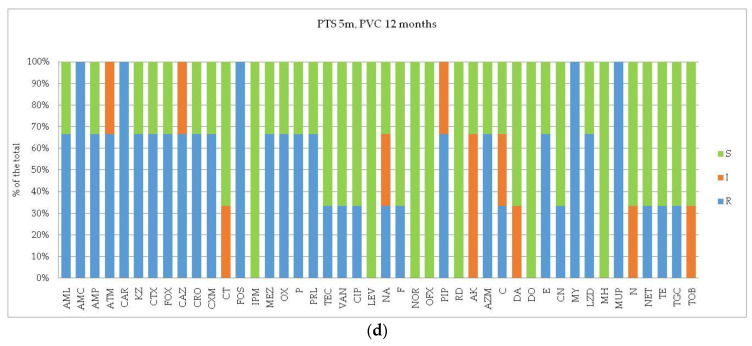
(**a**–**d**) Road Bay area. Results of antibioticsusceptibility tests of bacterial strains isolated from PVC-associated biofilm. Per each assayed antibiotic, the resistance percentages on the total of the bacterial isolates were calculated. The different panels represent the profiles detected at the impact site (Road Bay, R, affected by anthropic pressure) versus the control site (Punta Stocchino, PTS, unaffected).

**Figure 4:**
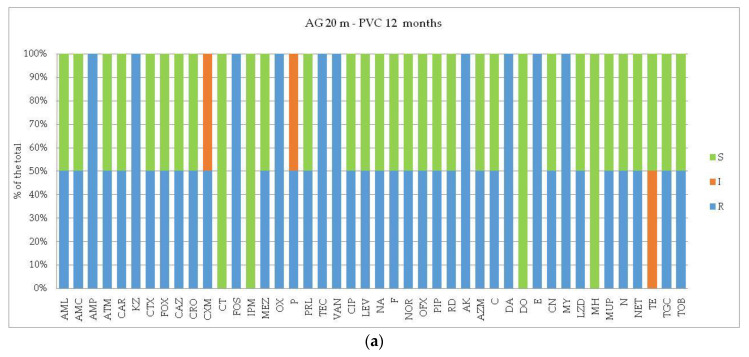

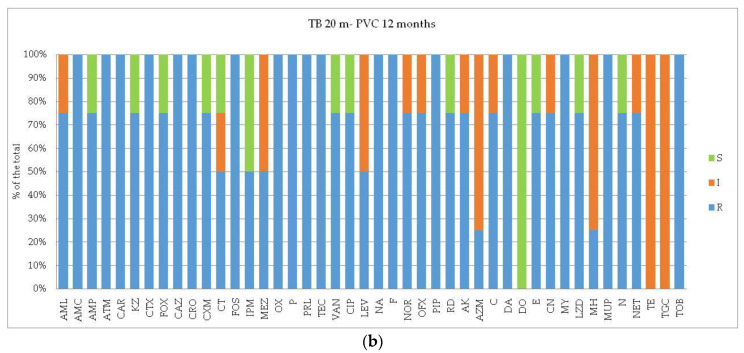
(**a**,**b**) Tethys Bay area. Results of antibioticsusceptibility tests of bacterial strains isolated from PVC-associated biofilm. Per each assayed antibiotic, the resistance percentages on the total of the bacterial isolates were calculated. The different panels represent the profiles detected at the impact site (Amorphous Glacier, AG, affected by a natural forcing such as a salinity gradient) versus the control site (Tethys Bay, TB).

**Figure 5:**
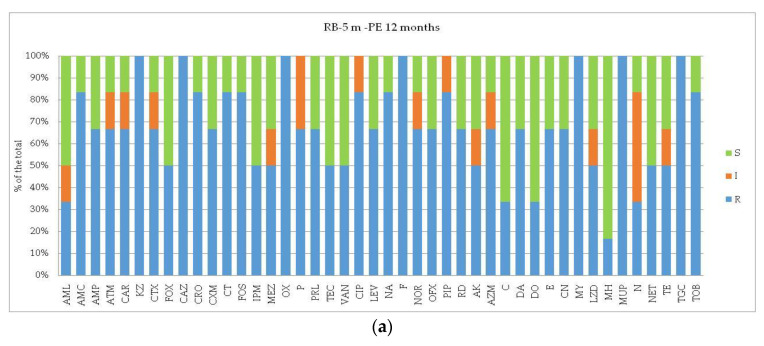

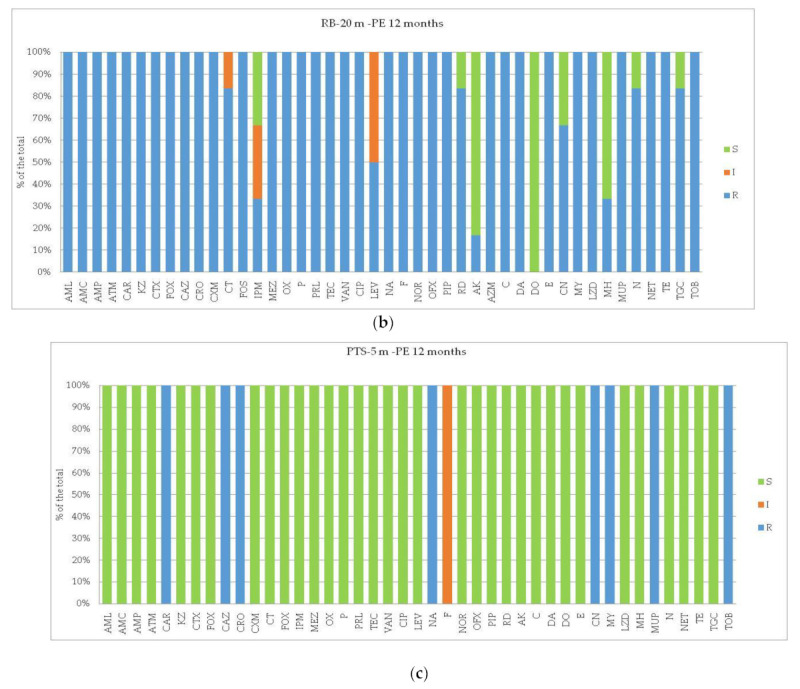
(**a**–**c**) Road Bay area. Results of antibioticsusceptibility tests of bacterial strains isolated from PE-associated biofilm. Per each assayed antibiotic, the resistance percentages of the total of the bacterial isolates were calculated. The different panels represent the profiles detected at the impact site (Road Bay, R, affected by anthropic pressure) versus the control site (Punta Stocchino, PTS, unaffected).

In detail, the profiles of susceptibility to each of the assayed antibiotics of the bacterial isolates are shown in Figure 3, Figure 4 and Figure 5 per each station and matrix separately.

In Road Bay (Figure 3), at station RB the PVC-associated plastisphere after 9 months of immersion showed peaks of ARB against P, AML, KZ, CTX, CRO, CXM, MY; with the increase inthe immersion period, the percentage of ARB increased for CAR, KZ, CTX, CRO, CXM, OX, CT, NA, PIP, AZM, MY, and in addition, new resistance was detected against VAN, TE, and TGC.

At station PTS, the high percentage of ARB found within the PVCplastisphere growing after 9 months was mostly attributed to antibiotics involved in the cell wall such as AML, AMC, AMP, FOX, OX, P. TEC, VAN as well as to inhibitors of protein synthesis including LZD, MUP, N, TGC, TOB, and AK, AZM, E, MY. After 12 months, also new antibiotic resistances were found (ATM, CRO, PRL, CIP, NA, PIP, NET, and TE).

In Tethys Bay (Figure 4), at the impact station AG, the PVC-associated plastisphere displayed full resistance against AMP, KZ, FOS, OX, TEC, VAN as well as against AK, DA, E, MY. Conversely, bacterial isolates were fully sensitive to CT, IPM, DO, and MH. At the control station TB, the incidence of ARB increased, with respect to the percentages of resistance to ATM, AMC, CAR, CAZ, CRO, as well as to P and PRL. Resistance to NA, PIP, F, and MUP and TOB increased too. Intermediate resistant strains to AZM, AK, C, MH, TE, and TGC were also recorded.

In the strains isolated from the PE-associated plastisphere from Road Bay (Table 2 and Figure 5), higher mean percentages of ARB were observed at RB station compared to PTS station, particularly at a 20 m depth. Conversely, at PTS station the percentage values of bacteria sensitive to the assayed antibiotics almost doubled compared to those measured in the strains isolated from RB station. On the PE panels submerged for 12 months at RB station, a high percentage of ARB reaching values of 67.43 and 89.01% of the total was recorded in the bacterial strains isolated from 5 and 20 m, respectively. On the other hand, at the PTS control site, not exposed to sewage effluent impact, and at a shallower depth (5 m), the bacterial strains isolated from the PE-associated plastisphere were found to be almost fully susceptible (77.27% of the total) to the assayed antibiotics.

With respect to each assayed antibiotic, the profiles of susceptibility yielded by the strains isolated from RB station from PE-associated biofilms (Figure 5) pointed out the occurrence of bacteria almost fully resistant to all the three classes of antibiotics, especially at 20 m, with the exception of AK, DO, and MH to which the strains were sensitive. Conversely, the bacterial isolates from PTS station were characterized by a wide susceptibility to the assayed antibiotics, with multiple-resistant bacteria against CAR, CRO, CAZ, FOS, CN, MY, MUP, NA, and TOB only.

In comparison with the biofilm samples, the average percentages of R, I, and S strains isolated from the surrounding seawater samples are reported in Table 3. The bacterial strains isolated from seawater all showed high percentages of antibiotic resistance, suggesting that a natural resistome also exists in the Antarctic environment, i.e., even in environments apparently not polluted by antibiotics. The average percentage of ARB ranged between 62.70 (RB-20 m) and 72.20% (TB-20 m) of the total, while that of bacteria sensitive to antibiotics was included, in the same sites, between 24.10% and 17.00%.

**Figure 6:**
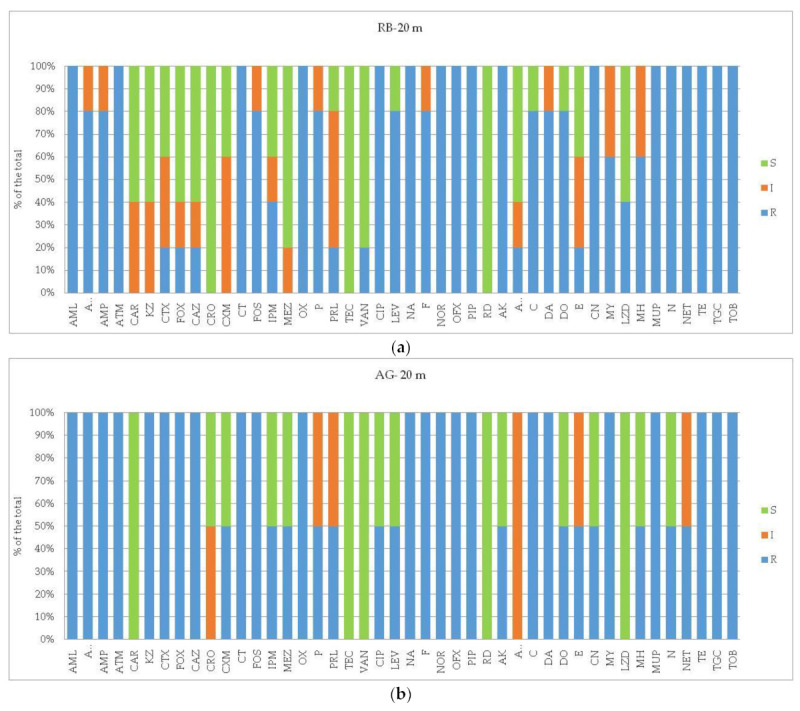

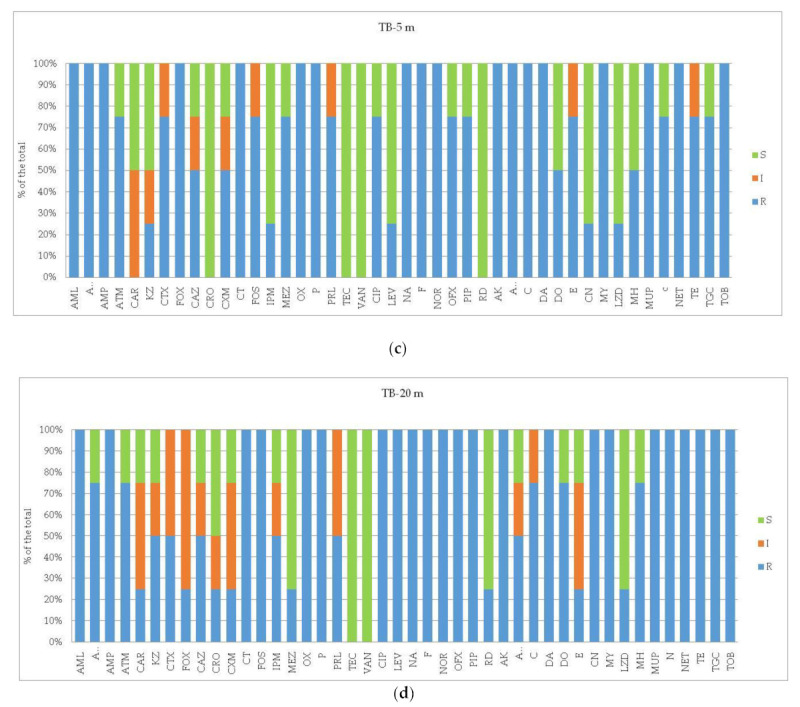
(**a**–**d**) Results of antibioticsusceptibility tests of bacterial strains isolated from water samples. Per each assayed antibiotic, the resistance percentages on the total of the bacterial isolates were calculated. The different panels represent the profiles detected at the impact site in Road Bay (RB) and at both sites in Tethys Bay (Amorphous Glacier, AG, versus its control site Tethys Bay, TB).

The antibiotic susceptibility assays carried out on the bacterial strains isolated from the water samples yielded the profiles shown in Figure 6. Water isolates from station TB-20 m exhibited multiple resistances to all the assayed antibiotics, with a percentage higher at the 5 m depth, with the exception of TEC and VAN which the isolates were sensitive to. At station RB-20 m, sensitive strains to CAR, KZ, FOX; CAZ, CRO, MEX, TEC, VAN, and RD were also detected.

### 3.3. 16S rRNA Sequencing of Bacterial Isolates

A total of 21 bacterial strains, chosen amongst the most representative of those isolated from the three different matrices (water, PVC-associated and PE-associated biofilms) were identified by 16S rRNA sequencing.The outputs of 16S rRNA sequencing are reported in Table 4. While Gammaproteobacteria predominated in all the matrices, plastisphere hosted a bacterial community different for composition from that occurring in seawater. From seawater samples, *Planococcus* and *Pseudoalteromonas* strains were isolated.On PVC, the plastisphere hosted a more diversified taxonomic composition at PTS station (control site) than at the RB one, suggesting the occurrence of a specialized flora in sites potentially exposed to pollution sources. On PE, bacterial isolates were identified to belong to the genus *Psychrobacter* at the RB site and *Pseudoalteromonas* at the PTS site.

### 3.4. MAR Index

To study the spread of antibiotic resistance in the study area, the MAR (Multiple Antibiotic Resistance) index was calculated. This is given by the formula

**Figure 7:**
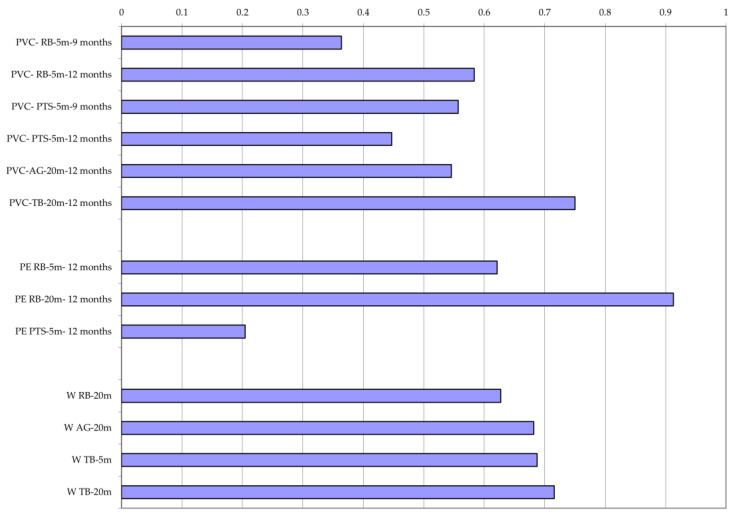
Multiple Antibiotic Resistance (MAR) index calculated from the results of antibioticsusceptibility assay of bacterial strains isolated from biofilm and water samples. PVC, polyvinylchloride; PE, polyethylene; W, water. Road Bay area: impact site (RB, Road Bay). Tethys Bay area: control (TB, Tethys Bay) and impact (AG, Amorphous Glacier).

“a/b”(1)

where “a” is the number of antibiotics tested to which each bacterial strain was resistant and “b” is the total number of antibiotics against which each single bacterial strain was tested [27]. The MAR index values per each matrix and station (Figure 7) reflected the number of ARB. For the water matrix, the MAR index reached higher values at the station TB-20 m, while lower values were observed at station RB-20 m. Regarding the plastisphere matrix, PE-associated biofilm bacteria showed MAR values generally higher than those found on PVC, with the exception of the station PTS-5 m where the minimum value of MAR was calculated. For the biofilm bacteria developed on PVC, higher MAR index values were observed at the TB-20 m station, while lower ones were calculated for the remaining samples.

By calculating the Pearson’s correlation coefficients *r* between the MAR index and the percentage of resistance per each category of antibiotics, a significant positive correlation was observed in water between MAR and lincosamides as well as nitrofurans (r = 0.915) (Table 5). This result suggests that lincosamides and nitrofurans are responsible for 91.5% of the variance of the MAR index.

In biofilm samples, Pearson’s correlations between MAR and the percentage of resistance per antibiotics’ category were lower than those calculated for water samples, but the number of significant values suggested that the biofilm matrix favored the incidence of a higher percentage of ARB compared to water. In PVC-associated biofilm bacteria, significant correlations were observed for quinolones, lincosamides, and rifamycins, antibiotic molecules that contributed to 85.7, 81.7, and 82.9% of the variance in the MAR index, respectively. In PE-associated biofilm bacteria, the correlations reached values equal to r = 0.99 for molecules such as beta-lactams, cephalosporins, oxazolidinones, and glycopeptides.

## 4. Discussion

Plastic pollution is increasing its occurrence on extreme polar environments and biota (see [7,8] as reviews);nevertheless, in remote regions important aspects such as those regarding the spread of ARB, with potential implications for human and animal health, need to be explored in greater detail. Particularly, the links among bacterial colonization via biofilm production, pollutants, and the resistome remain under-investigated. The present study is a first contribution on these topics. Plastics have been shown to host unique biofilm microbial communities, including bacterial pathogens [28,29], and to provide a suitable vector for the transfer of ARB and ARGs across both aquatic and terrestrial environments, posing potential environmental and public health risks [30,31,32].

It must be noted that the marine biofouling process through the formation of microbial biofilms can affect the interactions of plastics’ surface with adsorbed pollutants. Indeed, biofilms can modify the properties of plastic polymers, favoring their vertical transport, biodegradation process, and gene transmission via horizontal transfer mechanisms [33]. Their effects, however, may be different in relation to the biofilm composition and plastic and pollutants’ type [33]; for example, on high-density PE, biofilm was found to enhance significantly the sorption capacity of organic pollutants [34].

Among plastic polymers, PVC and PE are two materials that found wide applications in domestic and industrial fields.PVC is a synthetic material containing chlorine in its molecule, characterized by a strong and rigid structure, high chemical stability to acids and alkalis, resistance to aging and flame retardants, used for plastic wrap and cables. PE is a non-toxic polymer of ethylene;it is a polyolefin with a very low fragility, with high resistance to cold and radiations, used for food packaging, utensils, and in the electronics industry. Because of their structure, the interaction of these materials with the microbial assemblage could represent a significant concern, as both could serve as vectors for ARB [1,4].

This study aimed at assessing in the Ross Sea the antibiotic susceptibility profiles of bacterial strains isolated from the plastisphere colonizing PVC and PE panels and the surrounding seawater, in order to obtaininsights on the spread of ARB in two Antarctic sites.

### 4.1. Biofilm Formation

All the Antarctic strains isolated from the plastisphere and seawater matrices were able to produce biofilm, even if low OD values were frequently recorded. Globally, the low OD values recorded in our study could be consistent with the production of anti-biofilm molecules found in some Antarctic microorganisms [35]. In the examined strains, the biofilm production did not seem to be significantly affected by the source type (plastisphere or water). Conversely, the variable depth resulted in a higher ability to produce biofilm, as found in the bacteria isolated from 20 m compared to those isolated from 5 m. Although no significant differences in the biofilm production were found in relation to the study sites, the strains isolated from the impact station RB were more active in producing biofilms than those isolated from the other stations, suggesting that the aggregation of bacteria into a biofilm matrix could be a strategy for cold-adapted bacteria enabling them to increase their chances of survival in a potentially impacted site. Biofilm formation is affected by several environmental factors, among which temperature plays a critical role; although Antarctic bacterial communities can be structured in biofilms, the adaptation mechanisms though which this lifestyle is linked to the hosting environment are still mostly unknown [36].

### 4.2. Susceptibility to Antibiotics of the Bacterial Isolates

Screening of susceptibility to antibiotics of the plastisphere isolates underlined that, within the PVC-associated plastisphere collected from the impact station RB-5 m, the average percentages of ARB increased from 50.38 to 65.90% with the increase inthe immersion period from 9 to 12 months. Conversely, the percentage of resistance decreased over time at the control station PTS-5 m, suggesting that the different profiles of antibiotics’ susceptibility could depend on the different environmental conditions that indirectly shape the microbial communities hosted in the studied sites. In our study, the widespread detection of bacterial strains displaying multiple antibiotic resistance (from both plastisphere and seawater), especially at station RB close to the Italian research base, suggested that human presence was a source of AMR. However, the isolation of multidrug-resistant bacteria also from the Tethys Bay and Amorphous Glacier areas, not subject to anthropogenic forcing, suggested the presence in the studied Antarctic marine environment of a natural resistome. This finding agreed with previous studies in which the presence of a resistome was found in Antarctic soils apparently not exposed to contamination by antibiotics [15].

Grouping the susceptibility response of bacterial strains per each category of antibiotics, in bacteria isolated from biofilms associated to PVC and PE, both at control and impact sites, regardless of the depth, full resistance to antibiotics targeting cell wall was detected; conversely, bacteria showed 100% sensitivity against inhibitors of protein synthesis such as Doxycycline (DO) and Minocycline (MH) belonging to the tetracyclines group. In the isolates from seawater, although the greatest resistance percentages were observed against both antibiotics targeting the cell wall and those inhibiting protein synthesis, full sensitivity was found against antibiotics that inhibit cell wallsynthesis.

Within microbial biofilms, high numbers of metabolically active cells coexist, sharing a limited space; the complex architecture and the exopolysaccharidic matrix of biofilms can favor the transfer of resistance genes, including those for antibiotics and heavy metals [18,37,38,39,40]. Biofilms on plastics are considered as hotspots for horizontal gene transfer due to high cell density and nutrient availability [41]; besides gene transfer, other mechanisms involved in the spread of ARB are the exposure of bacteria to antibiotics adsorbed on the plastic surface or the co-selection of plastic/metals resistance. Within the plastisphere community, mechanisms of selection for AMR were also observed [42].

The sorption of antibiotics onto plastics surface has been addressed by a few studies only [43,44,45]. The potential adsorption capacity was reported to depend on the type of plastics; for tetracycline, this was to be in the order PE > polystyrene > PVC [45]. Also, the aging of microplastics, resulting in the formation of carbonyl groups, was found to increase the adsorption capacity of sulfamethoxazole to microplastics [43]. Adsorption of ciprofloxacin on PE has been reported to result in the outbreak of antibiotic-resistance phenomena across the environment [44].

In psychrophilic bacteria, resistance to ampicillin, chloramphenicol, kanamycin, and streptomycin was reported [46,47,48,49]. In Antarctic environments, previous studies reported on the isolation of ARB from different matrices, including seawater, sediments, and marine biota [16,47,48,49,50,51], where antibiotic resistance occurred frequently in association with heavy metal resistance. Recently, ARGs have been found in Antarctic soil, snow, and water samples [11,52,53,54,55,56], as well as in ice glaciers, previously thought to be pristine environments [57]. First observations of ARB date back to Miller et al. [47], who at the Palmer Station (Western Antarctic Peninsula) found increased numbers of multidrug-resistant bacteria near the research station. Similarly, Hernández and González-Acuña [58] documented that antibiotic-resistant human pathogens were detected in proximity to Antarctic research stations in relation to the anthropic presence. In wastewater and seawater from King George Island (South Shetland Islands), bacteria resistant to quinolones and trimethoprim were found in close association with the detection of antibiotics [49], proving that the anthropic impact generated by the scientific and tourism activities affected significantly the natural microbial resistome in the area. Surprisingly, multidrug-resistant bacteria were isolated also from a site such as Deception Island (Maritime Antarctica) not significantly affected by human activity [49]; there, multiple resistance to at least three or more antibiotics was observed in most of the bacterial isolates, which belonged to *Pseudomonas* spp. and Microbacteriaceae. In the Northern Antarctic Peninsula, González-Alonso et al. [5] reported on the presence of pharmaceuticals (e.g., analgesics, anti-inflammatory drugs, and antibiotics) and drugs in different freshwater bodies (streams, ponds, a glacier drain, and wastewater discharge).

### 4.3. Identification of ARB Isolated from Plastisphere and Water Samples

Most of the bacterial strains isolated from the PVC- and PE-associated plastispheres in our study were identified to be *Psychrobacter* spp. A strain of *P. psychrophilus* MR29-12 was isolated from an Eastern Siberian Sea permafrost [59], carrying a plasmid involved in the resistance to both streptomycin and tetracycline. Furthermore, the detection of bacterial strains belonging to the genus *Pseudoalteromonas*, which is a hydrocarbon-degrading bacterium, allows us to suppose its involvement in plastic decomposition. Members of the genera *Flavobacterium*, *Pseudomonas*, and *Desulfovibrio* isolated from the plastisphere were reported to host multiple ARGs [38,60]. Within micro- and mesoplastics-associated biofilms collected from King George Island, strains of *Pseudoalteromonas* and *Shewanella* spp. showed multiple resistance to cephalosporins, quinolones, and beta-lactams [18].

### 4.4. Potential Implication of ARB on Human and Ecosystem Health

ARB can persist in soil and water, and from these contaminated environmental sources they can spread via direct or indirect contact to humans and animals. ARB can transfer resistance to other bacteria via horizontal gene transfer mechanisms and forming multidrug-resistant bacteria [61]. They represent a well-known risk to human health, making ineffective the treatment with antibiotics of infectious diseases and causing socio-economic problems, so becoming one of the most urgent public health threats [62]. The occurrence of ARB in aquatic environments represents a significant threat not only to human health but also to the environment, breaking the equilibria and altering the resilience capability of the ecosystem and consequently the health of aquatic organisms [63]. When the equilibria within microbial communities are altered, both nutrient cycling and ecosystem stability are damaged and, therefore, also the welfare of the biota living within these systems may be significantly altered [64,65]. Indeed, ARB can impact on wildlife including several vertebrate organisms, from birds to reptiles; their presence is affected by multiple factors, including habitat, feeding, and general behavior of the animal species [66].

More studies are however necessary to better understand the ecological risks related to the spread of ARB within the microbial communities colonizing plastics in the marine environment [11]; especially in polar regions, there is a knowledge gap on this topic [55]; therefore, our study is a first contributionto assess the role of plastic polymers as a reservoir of ARB under extreme environmental conditions.

## 5. Conclusions

Studying the diversity and abundance of ARB in regions characterized by relatively low anthropogenic activity like Antarctica may provide valuable insights into the mechanism controlling the evolution of antibiotic resistance. The findings of our study point out that in the Ross Sea no selective enrichment of ARB occurred in relation to study sites or to either type of plastic material, suggesting that resistance to antibiotics was a generalized widespread phenomenon, in agreement with previous studies [54]. Resistance against all the three classes of antibiotics assayed in this study (i.e., cell wall antibiotics, nucleic acids, and protein synthesis inhibitors) was observed. Multidrug-resistant bacteria against all the three classes of antibiotics assayed in this study (i.e., cell wall antibiotics, nucleic acids, and protein synthesis inhibitors) was recorded. The high resistance percentages found in the bacterial strains isolated from both biofilm and seawater samples stress the need to pay more attention to understanding the interactions between plastics, antibiotic residues, and microbial biofilms, as underlined by recent reports [61]. Given the emerging occurrence of plastics in this remote region, the detection of superbug bacteria in the Ross Sea not only through plastic items but also in the aquatic medium could reveal a small part only of a threat whose actual entity and related risks to human/animals’ health remains to be discovered yet, namely like the tip of an iceberg.

## Figures and Tables

**Table 1:** Conversion factors of the antibiogram results into arbitrary quantitative values.

Qualitative Result	Quantitative Value
R (−−−−; +−−−)	0
I (++−−; +++−)	1
S (++++)	2
SS (+++++)	3

where R = resistant, I = moderately sensitive, S=sensitive, and SS=very sensitive. The + and − indicate the degree of reaction intensity.

**Table 2:** Mean percentages of resistant (R), intermediate resistant (I), and susceptible (S) strains of the total bacteria isolated from biofilm samples associated to PVC and PE panels. PVC = Poly Vinyl Chloride; PE = Polyethylene. Road Bay: control (PTS, Punta Stocchino) and impact (RB, Road Bay). Tethys Bay: control (TB, Tethys Bay) and impact (AG, Amorphous Glacier).

PVC	Months	R	I	S	R+I
RB-5 m	9	36.74	13.64	49.62	50.38
RB-5 m	12	56.80	9.10	34.10	65.90
PTS-5 m	9	55.70	21.60	22.70	77.30
PTS-5 m	12	44.70	8.30	47.00	53.00
AG-20 m	12	55.68	3.41	40.91	59.09
TB-20 m	12	75.57	14.77	9.66	90.34
**PE**	**Months**	**R**	**I**	**S**	**R+I**
RB-5 m	12	67.43	6.43	26.14	73.86
RB-20 m	12	89.01	2.27	8.72	91.28
PTS-5 m	12	20.46	2.27	77.27	22.73

**Table 3:** Average percentages of resistant (R), intermediate resistant (I), and susceptible strains (S) of the total bacteria isolated from the Ross Sea water samples. RB, Road Bay (impact site); TB, Tethys Bay (control site); AG, Amorphous Glacier (impact site), at 5 and 20 m depths.

	R	I	S	R+I
RB-20 m	62.70	13.20	24.10	75.90
TB-5 m	68.80	5.70	25.50	74.50
TB-20 m	72.20	10.80	17.00	83.00
AG-20 m	68.20	6.80	25.00	75.00

**Table 4:** 16S rRNA sequencing of the bacterial strains isolated from the biofilm and water samples. Per each isolate are reported the matrix (polyvinylchloride (PVC), polyethylene, (PE), or water), the site of provenance and duration of immersion (for biofilm microbes only), the identification with indication of the Class, closest relative and percentage of similarity, and GenBank accession number.

Matrix	Source	Immersion Months	Class	Identification	Similarity %	Closest Relative	GenBank Accession Number
PVC	RB-5 m	9	Gammaprot.	*Psychrobacter luti*	99.9	KJ475194	OQ547277
PVC	RB-5 m	12	Gammaprot.	*Pseudoalteromonas* sp.	99.91	MN889217	OQ547278
PVC	RB-5 m	12	Gammaprot.	*Psychrobacter nivimaris*	100	MN062081	OQ547288
PVC	PTS-5 m	9	Gammaprot.	*Psychrobacter glacincola*	99.8	AB681354	OQ547285
PVC	PTS-5 m	9	Gammaprot.	*Pseudomonas* sp.	100	MT585904	OQ547294
PVC	PTS-5 m	12	Bacteroidota	*Winogradskyella* sp.	97.79	MK780011	OQ547275
PVC	PTS-5 m	12	Gammaprot.	*Pseudoalteromonas* sp.	99.8	MN889217	OQ547276
PVC	PTS-5 m	12	Gammaprot.	*Psychrobacter glacincola*	100	AB681354	OQ547293
PVC	TB-20 m	12	Actinobacteria	*Arthrobacter* sp.	99.9	MK660306	OQ547280
PVC	AG-20 m	12	Gammaprot.	*Psychrobacter glacincola*	100	AB681354	OQ547283
PVC	AG-20 m	12	Gammaprot.	*Psychrobacter glacincola*	99.8	AB681354	OQ547284
PE	RB-5 m	12	Gammaprot.	*Psychrobacter fozii*	100	KX417150	OQ547279
PE	RB-5 m	12	Actinobacteria	*Arthrobacter flavus*	99.79	MF541537	OQ547281
PE	RB-20 m	12	Gammaprot.	*Psychrobacter glacincola*	100	AB681354	OQ547287
PE	RB-20 m	12	Gammaprot.	*Psychrobacter* sp.	99.9	MK560045	OQ547282
PE	RB-20 m	12	Gammaprot.	*Psychrobacter* sp.	100	KT989022	OQ547286
PE	RB-20 m	12	uncultured bacterium	*Psychrobacter* sp.	97.29	JQ191298	OQ547292
PE	PTS-5 m	12	Gammaprot.	*Pseudoalteromonas mariniglutinosa*	100	KP236350	OQ547274
Water	RB-20 m		Firmicutes	*Planococcus halocryophilus*	100	NR118149	OQ547291
Water	AG-5 m		Gammaprot.	*Pseudoalteromonas* sp.	99.31	MG388174	OQ547289
Water	AG-20 m		Gammaprot.	*Pseudoalteromonas arctica*	99.81	KY508316	OQ547290

**Table 5:** Pearson’s correlation coefficients computed between the MAR index and the percentage of resistance per each category of antibiotics.

	Biofilm PVC	Biofilm PE	Water
Beta-lactams	0.787	0.993	0.065
Macrolides	0.200	0.996	0.442
Quinolones	0.857	0.999	−0.097
Aminoglycosides	0.760	0.965	−0.174
Cephalosporin	0.286	0.988	0.618
Lincosamides	*0.817*	0.996	0.915
Chloramphenicol	0.611	0.957	0.049
Cyclic polypeptides	0.142	0.912	0.143
Tetracycline	0.192	0.938	−0.121
Fosfomycin	0.405	−0.101	0.262
Oxazolidinones	0.607	0.994	−0.464
Nitrofurans	0.763	0.912	0.915
Rifamycin	*0.829*	0.973	0.680
Glycopeptides	0.753	0.995	−0.915

Statistically significant values at *p* < 0.01 are reported in bold; at *p* < 0.05 in italics.

## Data Availability

The raw data supporting the conclusions of this article will be made available by the authors on request. Data have been deposited at the PNRA website, Italian Antarctic Data Center (https://iandc.pnra.aq/srv/ita/catalog.search#/home (accessed on 17 March 2023)).

## References

[1] Caruso G. (2020). Microbial Colonization in Marine Environments: Overview of Current Knowledge and Emerging Research Topics. J. Mar. Sci. Eng..

[2] Zhai X., Zhang X.H., Yu M. (2023). Microbial colonization and degradation of marine microplastics in the plastisphere: A review. Front. Microbiol..

[3] Caruso G. (2019). Microplastics as vectors of contaminants. Mar. Pollut. Bull..

[4] Corsolini S. (2009). Industrial contaminants in Antarctic biota. J. Chromatogr. A.

[5] González-Alonso S., Merino L.M., Esteban S., López de Alda M., Barceló D., Durán J.J., López-Martínez J., Aceña J., Pérez S., Mastroianni N. (2017). Occurrence of pharmaceutical, recreational and psychotropic drug residues in surface water on the northern Antarctic Peninsula region. Environ. Pollut..

[6] Szopińska M., Potapowicz J., Jankowska K., Luczkiewicz A., Svahn O., Björklund E., Nannou C., Lambropoulou D., Polkowska Z. (2022). Pharmaceuticals and other contaminants of emerging concern in Admiralty Bay as a result of untreated wastewater discharge: Status and possible environmental consequences. Sci. Total Environ..

[7] Caruso G., Bergami E., Singh N., Corsi I. (2022). Plastic occurrence, sources, and impacts in Antarctic environment and biota. Water Biol. Secur..

[8] Rota E., Bergami E., Corsi I., Bargagli R. (2022). Macro- and Microplastics in the Antarctic Environment: Ongoing Assessment and Perspectives. Environments.

[9] Zhuang S., Wang J. (2023). Interaction between antibiotics and microplastics: Recent advances and perspective. Sci. Total Environ..

[10] Xie S., Hamid N., Zhang T., Zhang Z., Peng L. (2024). Unraveling the nexus: Microplastics, antibiotics, and ARGs interactions, threats and control in aquaculture—A review. J. Hazard. Mater..

[11] Yang Y., Liu G., Song W., Ye C., Lin H., Li Z., Liu W. (2019). Plastics in the marine environment are reservoirs for antibiotic and metal resistance genes. Environ. Int..

[12] World Health Organization (WHO) (2022). Global Antimicrobial Resistance and Use Surveillance System (GLASS).

[13] Hwengwere K., Paramel Nair H., Hughes K.A., Peck L.S., Clark M.S., Walker C.A. (2022). Antimicrobial resistance in Antarctica: Is it still a pristine environment?. Microbiome.

[14] Surette M.D., Wright G.D. (2017). Lessons from the environmental antibiotic resistome. Ann. Rev. Microbiol..

[15] Van Goethem M.W., Pierneef R., Bezuidt O.K.I., Van De Peer Y., Cowan D.A., Makhalanyane T.P. (2018). A reservoir of ‘historical’ antibiotic resistance genes in remote pristine Antarctic soils. Microbiome.

[16] Lo Giudice A., Caruso G., Rizzo C., Papale M., Azzaro M. (2019). Bacterial communities versus anthropogenic disturbances in the Antarctic coastal marine environment. Environ. Sustain..

[17] Mah T.F. (2012). Biofilm-Specific Antibiotic Resistance. Future Microbiol..

[18] Laganà P., Caruso G., Corsi I., Bergami E., Venuti V., Majolino D., La Ferla R., Azzaro M., Cappello S. (2019). Do plastics serve as a possible vector for the spread of antibiotic resistance? First insights from bacteria associated to a polystyrene piece from King George Island (Antarctica). Int. J. Hyg. Environ. Health.

[19] Smith W.O., Ainley D.G., Arrigo K.R., Dinniman M.S. (2014). The Oceanography and Ecology of the Ross Sea. Annu. Rev. Mar. Sci..

[20] Halpern B.S., Walbridge S., Selkoe K.A., Kappel C.V., Micheli F., D’Agrosa C., Bruno J.F., Casey K.S., Ebert C., Fox H.E. (2008). A Global Map of Human Impact on Marine Ecosystems. Science.

[21] Caroppo C., Azzaro M., Dell’Acqua O., Azzaro F., Maimone G., Rappazzo A.C., Raffa F., Caruso G. (2022). Microbial biofilms colonizing plastic substrates in the Ross Sea (Antarctica). J. Mar. Sci. Eng..

[22] O’ Toole G., Kaplan H.B., Kolter R. (2000). Biofilm formation as microbial development. Annu. Rev. Microbiol..

[23] Caruso G., Dell’Acqua O., Caruso R., Azzaro M. (2022). Phenotypic characterization of bacterial isolates from marine waters and plastisphere communities of the Ross Sea (Antarctica). J. Clin. Microbiol. Biochem. Technol..

[24] Altschul S.F., Madden T.L., Schaffer A.A., Zhang J., Zhang Z., Miller W., Lipman D.J. (1997). Gapped BLAST and PSI-BLAST: A new generation of protein database search programs. Nucleic Acids Res..

[25] Bauer A.W., Kirby W.M., Sherris J.C., Turk M. (1966). Antibiotic susceptibility testing by a standardized single disk method. Am. J. Clin. Pathol..

[26] EUCAST (2018). European Committee on Antimicrobial Susceptibility Testing. Breakpoint Tables for Interpretation of MICs and Zone Diameters. Version 8.1. https://www.eucast.org/.

[27] Laganà P., Votano L., Caruso G., Azzaro M., Giudice A.L., Delia S. (2018). Bacterial isolates from the Arctic region (Pasvik River, Norway): Assessment of biofilm production and antibiotic susceptibility profiles. Environ. Sci. Pollut. Res..

[28] Cappello S., Caruso G., Bergami E., Macrì A., Venuti V., Majolino D., Corsi I. (2021). New insights into the structure and function of the prokaryotic communities colonizing plastic debris collected in King George Island (Antarctica): Preliminary observations from two plastic fragments. J. Hazard. Mater..

[29] Papale M., Fazi S., Severini M., Scarinci R., Dell’Acqua O., Azzaro M., Venuti V., Fazio B., Fazio E., Crupi V. (2024). Structural properties and microbial diversity of the biofilm colonizing plastic substrates in Terra Nova Bay (Antarctica). Sci. Total Environ..

[30] Jacquin J., Cheng J., Odobel C., Pandin C., Conan P., Pujo-Pay M., Barbe V., Meistertzheim A.-L., Ghiglione J.-F. (2019). Microbial ecotoxicology on marine plastic debris: A review on colonization and biodegradation by the “plastisphere”. Front. Microbiol..

[31] Perveen S., Pablos C., Reynolds K., Stanley S., Marugan J. (2022). Microplastics in fresh and wastewater are potential contributors to antibiotic resistance. A minireview. J. Hazard. Mater..

[32] Sooriyakumar P., Bolan N., Kumar M., Singh L., Yu Y., Li Y., Weralupitiya C., Vithanage M., Ramanayaka S., Sarkar B. (2022). Biofilm formation and its implications on the properties and fate of microplastics in aquatic environments: A review. J. Hazard. Mater. Adv..

[33] Ventura M., Marin A., Gàmez-Pérez J., Cabedo L. (2024). Recent advances in the relationships between biofilms and microplastics in natural environments. World J. Microbiol. Biotechnol..

[34] Cui W., Hale R.C., Huang Y., Zhou F., Wu Y., Liang X., Liu Y., Tan H., Chen D. (2023). Sorption of representative organic contaminants on microplastics: Effects of chemical physicochemical properties, particle size, and biofilm presence. Ecotoxicol. Environ. Saf..

[35] Artini M., Papa R., Vrenna G., Trecca M., Paris I., D’Angelo C., Tutino M.L., Parrilli E., Selan L. (2023). Antarctic Marine Bacteria as a Source of Anti-Biofilm Molecules to Combat ESKAPE Pathogens. Antibiotics.

[36] Parrilli E., Tutino M.L., Marino G. (2022). Biofilm as an adaptation strategy to extreme conditions. Rend. Fis. Acc. Lincei.

[37] Balcazar J.L., Subirats J., Borrego C.M. (2015). The role of biofilms as environmental reservoirs of antibiotic resistance. Front. Microbiol..

[38] Sun Y., Wang J. (2022). How microplastics and nanoplastics shape antibiotic resistance?. Water Emerg. Contam. Nanoplastics.

[39] Moyal J., Dave P.H., Wu M., Karimpour S., Brar S.K., Zhong H., Kwong R.W.M. (2023). Impacts of biofilm formation on the physicochemical properties and toxicity of microplastics: A concise review. Rev. Environ. Contam. Toxicol..

[40] Wang Z., Gao J., Zhao Y., Dai H., Jia J., Zhang D. (2021). Plastisphere enrich antibiotic resistance genes and potential pathogenic bacteria in sewage with pharmaceuticals. Sci. Total Environ..

[41] Mammo F.K., Amoah I.D., Gani K.M., Pillay L., Ratha S.K., Bux F., Kumari S. (2020). Microplastics in the environment: Interactions with microbes and chemical contaminants. Sci. Total Environ..

[42] Stevenson E.M., Buckling A., Cole M., Lindeque P.K., Murray A.K. (2023). Selection for antimicrobial resistance in the plastisphere. Sci. Total Environ..

[43] Guo X., Liu Y., Wang J. (2019). Sorption of sulfamethazine onto different types of microplastics: A combined experimental and molecular dynamics simulation study. Mar. Pollut. Bull..

[44] Atugoda T., Wijesekara H., Werellagama D.R.I.B., Jinadasa K.B.S.N., Bolan N.S., Vithanage M. (2020). Adsorptive interaction of antibiotic ciprofloxacin on polyethylene microplastics: Implications for vector transport in water. Environ. Technol. Innov..

[45] Yu F., Yang C., Huang G., Zhou T., Zhao Y., Ma J. (2020). Interfacial interaction between diverse microplastics and tetracycline by adsorption in an aqueous solution. Sci. Total Environ..

[46] De Souza M.J., Nair S., Loka Bharathi P.A., Chandramohan D. (2006). Metal and antibiotic-resistance in psychrotrophic bacteria from Antarctic Marine waters. Ecotoxicology.

[47] Miller R.V., Gammon K., Day M.J. (2009). Antibiotic resistance among bacteria isolated from seawater and penguin fecal samples collected near Palmer Station, Antarctica. Can. J. Microbiol..

[48] González-Aravena M., Urtubia R., Del Campo K., Lavín P., Wong C.M.V.L., Cárdenas C.A., González-Rocha G. (2016). Antibiotic and metal resistance of cultivable bacteria in the Antarctic sea urchin. Antarct. Sci..

[49] Hernández F., Calısto-Ulloa N., Gómez-Fuentes C., Gómez M., Ferrer J., González-Rocha G., Bello-Toledo H., Botero-Coy A.M., Boıx C., Ibáñez M. (2019). Occurrence of antibiotics and bacterial resistance in wastewater and sea water from the Antarctic. J. Hazard. Mater..

[50] Na G., Zhang W., Gao H., Wang C., Li R., Zhao F., Zhang K., Hou C. (2021). Occurrence and antibacterial resistance of culturable antibiotic-resistant bacteria in the Fildes Peninsula, Antarctica. Mar. Pollut. Bull..

[51] Calisto N., Navarro L., Orellana P., Wiese G., Gómez C., Salazar L., Cortés-Cortés P., Gutiérrez-Moraga A., Gidekel M., Corsini G. (2021). Resistencia a antibióticos y actividad antimicrobiana de aislados bacterianos de suelo antártico. AIP.

[52] Morozova O.V., Andreeva I.S., Zhirakovskiy V.Y., Pechurkina N.I., Puchkova L.I., Saranina I.V., Emelyanova E.K., Kamynina T.P. (2022). Antibiotic resistance and cold adaptive enzymes of antarctic culturable bacteria from King George Island. Polar Sci..

[53] Jara D., Bello-Toledo H., Domínguez M., Cigarroa C., Fernández P., Vergara L., Quezada-Aguiluz M., Opazo-Capurro A., Lima C.A., González-Rocha G. (2020). Antibiotic resistance in bacterial isolates from freshwater samples in Fildes Peninsula, King George Island, Antarctica. Sci. Rep..

[54] Marcoleta A.E., Arros P., Varas M.A., Costa J., Rojas-Salgado J., Berríos-Pastén C., Tapia-Fuentes S., Silva D., Fierro J., Canales N. (2022). The highly diverse Antarctic Peninsula soil microbiota as a source of novel resistance genes. Sci. Total Environ..

[55] Depta J., Niedźwiedzka-Rystwej P. (2023). The Phenomenon of Antibiotic Resistance in the Polar Regions: An Overview of the Global Problem. Infect. Drug Resist..

[56] Tamang S., Sharma P., Kumar S., Thakur N. (2024). Bacterial community structure, adaptations and prevalence of antimicrobial resistance in bacteria from Antarctica: A review. Polar Sci..

[57] Hernández J., González-Acuña D. (2016). Anthropogenic antibiotic resistance genes mobilization to the polar regions. Infect. Ecol. Epidemiol..

[58] Tam H.K., Wong C.M.V.L., Yong S.T., Blamey J., González M. (2015). Multiple-antibiotic-resistant bacteria from the maritime Antarctic. Polar Biol..

[59] Mindlin S.Z., Soina V.S., Petrova M.A., Gorlenko Z.M. (2008). Isolation of antibiotic resistance bacterial strains from Eastern Siberia permafrost sediments. Russ. J. Genet..

[60] Yang K., Chen Q.-L., Chen M.-L., Li H.-Z., Liao H., Pu Q., Zhu Y.-G., Cui L. (2020). Temporal Dynamics of Antibiotic Resistome in the Plastisphere during Microbial Colonization. Environ. Sci. Technol..

[61] Fajardo C., Sànchez-Fortùn S., Videira-Quintela D., Martin C., Nande M., D’ors A., Costa G., Guillen F., Montalvo G., Martin M. (2023). Biofilm formation on polyethylene microplastics and their role as transfer vector of emerging organic pollutants. Environ. Sci. Pollut. Res..

[62] Wang W., Weng Y., Luo T., Wang Q., Yang G., Jin Y. (2023). Antimicrobial and the Resistances in the Environment: Ecological and Health Risks, Influencing Factors, and Mitigation Strategies. Toxics.

[63] Ahmed S.K., Hussein S., Qurbani K., Ibrahim R.H., Fareeq A., Mahmood K.A., Mohamed M.G. (2024). Antimicrobial resistance: Impacts, challenges, and future prospects. J. Med. Surg. Public Health.

[64] Barathan M., Ng S.-L., Lokanathan Y., Ng M.H., Law J.X. (2024). Unseen Weapons: Bacterial Extracellular Vesicles and the Spread of Antibiotic Resistance in Aquatic Environments. Int. J. Mol. Sci..

[65] Monahan C., Nag R., Morris D., Cummins E. (2021). Antibiotic residues in the aquatic environment—Current perspective and risk considerations. J. Environ. Sci. Health A Tox. Hazard. Subst. Environ. Eng..

[66] Garcês A., Pires I. (2023). European Wild Carnivores and Antibiotic-Resistant Bacteria: A Review. Antibiotics.

